# Supplementary material for: Primary postpartum haemorrhage at the Libreville University Hospital Centre: Epidemiological profile of women
Source: PLoS One. 2021 Sep 20;16(9):e0257544. doi: 10.1371/journal.pone.0257544 (PMC8452036; doi:10.1371/journal.pone.0257544)
Supplement: Attachment — Submitted filename: Responses to Reviewers.DOCX [file pone.0257544.s001.DOCX]

**Response to Reviewers**

**Editor Comments**

1. We ensured that our manuscript meets PLOS ONE's style requirements, including those for file naming.
2. Additional details regarding participant consent were provided in the Methods and online submission information.

**Reviewer #**1

Thank you very much for the importance given to our manuscript. We find your comments very pertinent. We have taken these remarks into consideration and corrections have been made in the text.

**Abstract**

In fact we mean the proportion of maternal deaths related to IPPH. Using “birth weight˝ instead of child weight in the text. Repetition deleted

**Introduction**

We changed terms : using “primary˝ instead of “immediate˝ in the text

**Methods**

Brief background information on study area or setting, in relation to bed capacity, deliveries etc provided.

**Results**

We are talking here about a pathological gynecological history. Correction made

**Discussion**

We are talking here about delivery heamorrhage.

**Tables**

Correction made for Tables 1-3

**Additional comments**

Correction made. We used ‘tract’ insted of ‘track’ ; ‘post-term’ instead of ‘postterm’ through the manuscript. To avoid ambiguity, ‘N’= birth records (42,728) or study population and ‘n’= number of each item or variable.

**Reviewer #2**

Thank you very much for the importance given to our manuscript. We find your comments very pertinent. We have taken these remarks into consideration and corrections have been made in the text.

a) In our context we did not do a case control study
b) The obstetric practice was addressed as well as the cadre of operators but this is reported in another document. In fact during the study period, 42,728 birth records were collected, with an incidence of primary postpartum hemorrhage of 1.6% (n = 671). A total of 688 children, including 172 deaths, were born from mothers with PPPH.
c) Active Management of Third Stage of Labour (AMTSL) was not practiced in the University Hospital Centre of Libreville at the period of this study but is now practised. This could be a limitation of this study.
d) This question is related to the one asked in point C
e) This question is related to the one asked in point C
f) As suggested, it would be more appropriate to compare the active cases with a matched control for better understanding. We will consider this as a limitation of the study
g) This question is related to the one asked in point C
h) An English speaking writer helped us to verify the language and use of certain words in the text
